# The Relationship Between Pediatric Residents' Experiences Being Parented and Their Provision of Parenting Advice

**DOI:** 10.3389/fped.2018.00395

**Published:** 2018-12-12

**Authors:** Ami C. Bax, Paul M. Shawler, Michael P. Anderson, Mark L. Wolraich

**Affiliations:** ^1^Section on Developmental and Behavioral Pediatrics, Department of Pediatrics, Child Study Center, College of Medicine, University of Oklahoma Health Sciences Center, Oklahoma, OK, United States; ^2^Department of Biostatistics and Epidemiology, College of Public Health, University of Oklahoma Health Sciences Center, Oklahoma, OK, United States

**Keywords:** parenting, discipline, Pediatric residency training, pediatrics, primary care

## Abstract

**Background:** Factors surrounding pediatricians' parenting advice and training on parenting during residency have not been well studied. The Resident Parenting Questionnaire (RPQ) was developed to assess (a) the relationship between pediatric residents' upbringing and their parenting advice style and (b) factors associated with confidence and resource use when delivering parenting advice.

**Methods:** Three hundred and one pediatric residents from 15 United States residency programs completed the RPQ with upbringing and advice responses categorized using Baumrind's parenting model (authoritative, authoritarian, permissive). Chi-square/Fisher's exact tests, Bowker's test of symmetry, and regression analyses assessed associations between residents' upbringing, parenting advice style/content, and confidence in providing parenting advice.

**Results:** Most participants indicated being raised authoritatively (68%) and giving authoritative parenting advice (83%), but advice differed based on how they perceived their upbringing (*p* < 0.001). Residents noting authoritative upbringing were more likely to give authoritative advice (85%) while others tended to give advice differing from upbringing (e.g., those perceiving authoritarian upbringing were more likely to give authoritative/permissive). Analyses suggest resident race, acculturation, future plans, and resident level are associated with parenting advice type. Confidence in giving parenting advice decreased significantly as patient age increased and increased with resident level advancement. Residents reported consulting attending physicians for parenting advice guidance more than any other evidence-based resources.

**Conclusion:** Most pediatric residents appear to be aware of appropriate authoritative parenting advice regardless of upbringing, especially as they advance through residency. Residents may benefit from opportunities to reflect upon their upbringing, particularly if raised in authoritarian or permissive styles. Targeted training of residents on evidence-based parenting strategies, particularly for older pediatric patients, appears warranted.

## Introduction

For several decades, developmental and behavioral concerns have remained a sizeable portion of the complaints expressed in general pediatric visits. Despite a mandatory 1-month block rotation in developmental/behavioral pediatrics being implemented as a requirement during general pediatrics residency in 1997, pediatricians' comfort level with behavior problems remained unchanged a decade later (1). Furthermore, the 2009 American Academy of Pediatrics (AAP) Policy Statement clarifying the need for pediatricians to address mental health in primary care settings more effectively, many pediatric residencies fall short of preparing their residents to address their patients' behavioral and mental health needs as laid out by the AAP (2). In 2013, 65% of pediatricians surveyed by the AAP indicated that they lacked training in recognizing and treating mental health problems (3). Many positive child behavioral and mental health outcomes have been closely associated with balanced, authoritative parenting and discipline practices in numerous studies over the past 5 decades (4–6). Therefore, discussing parenting and discipline in pediatric visits provides an opportunity for pediatricians to identify and help address potential behavior problems from a young age, thus potentially preventing the development of future, more adverse child behavior problems.

Research has indeed found that pediatricians are frequently consulted for advice about parenting and discipline regarding their patients (7). The American Academy of Pediatrics (AAP) has noted that 90% of pediatricians report including advice about discipline when providing parental anticipatory guidance (8). While this has potential impact for positive parent behavior change, advice currently given by most pediatricians may be limited in scope and insufficient to effect change in the parent-child relationship (9). Further, only 23–43% of parents perceive that their pediatricians discuss discipline during visits (7, 10), raising additional questions about effectiveness of advice pediatricians provide.

Several aspects of pediatricians' parenting/discipline advice need further examination, including types of advice given, comfort addressing families' needs, and training/resources used during residency to formulate these recommendations. It is difficult to ascertain from the literature how many United States (U.S.) pediatric residency programs have existing formal curricula designed to enhance residents' ability to adequately address parenting/discipline, given that many programs do not necessarily publish or share their curricula. Upon our review, programs at Children's Mercy Hospital and Clinics (Kansas City), Floating Hospital for Children (Boston), and Children's Hospital at Oakland have all presented parenting curricula with some promising elements. However, only a few programs appear to have developed resident education curricula adapted from evidence-based parenting interventions [*Incredible Years* (11) at Indiana University and *Triple P Positive Parenting Program* (12) at Seattle Children's and Ohio] (13–15) and published studies establishing effectiveness of their curricula. No resident parenting curricula appear to have designed their program with an understanding of the pre-existing attitudes of pediatric residents.

To improve pediatricians' impact on parenting, it is important to examine resources residents use to provide parental advice about general child well-being. Without evidence-based training or curricula, it is plausible that residents' upbringing instead influence parenting advice as evidence suggests parenting behavior is passed through multiple generations (16). However, no medical or psychology studies have explored the relationship between clinicians' (e.g., physicians, psychologists, therapists) upbringing and parenting advice they give.

To investigate these issues, we assessed whether pediatric residents' type of parenting advice relates to their past experience, particularly if they are not taught about effective parenting during training. We used Baumrind's classic model (17), later expanded by Maccoby and Martin (18), to categorize both how residents perceived their own parents' parenting styles and their preferred parenting advice style in clinic. This model classifies highly demanding parenting with little responsiveness as authoritarian, highly responsive parenting with little demandingness as permissive, and parenting with balanced amounts of demandingness and responsiveness as authoritative (17, 18). Research has suggested that these three key parenting styles as defined by the Baumrind model are fairly evenly distributed in the general population with occasional predominance of authoritative parenting reported (19–24). Furthermore, promotion of authoritative parenting strategies are associated with positive outcomes in many evidence-based behavior modification parenting interventions such as *Parent Child Interaction Therapy* (25). Therefore, the Baumrind model was helpful in determining to what extent residents endorse authoritative versus other parenting strategies.

This study used survey methodology to examine the relationship between pediatric residents' upbringing and their parenting advice. Using this theoretical structure, we hypothesized that (a) similar to the general population, residents would vary in the perceived parenting styles they received as children, and (b) type of advice given to families would be associated with parenting type residents received as children. Secondary aims of the study were to explore residents' confidence and resources utilized in giving parenting advice.

## Materials and Methods

### Participants

All categorical and other residents at 15 of the 199 U.S. Accreditation Council for Graduate Medical Education pediatric residency programs (26) were invited to participate by completing the Resident Parenting Questionnaire (RPQ). Multisite survey administration spanned 2012–2014 with each program participating in waves lasting 3–4 weeks. Programs were recruited through the authors' connections with other residency program directors with an interest in improving parenting training or through the authors' affiliation with the Developmental & Behavioral Pediatric Research Network. Programs were also recruited to increase geographical and program variation and generalizability. The [institution name deleted to maintain integrity of review process] institutional review board approved the study.

### Questionnaire Development

The RPQ was developed to assess pediatric residents' self-perceived attitudes and skills in counseling families on effective parenting strategies. The measure was computerized, piloted, and iteratively refined through administration of preliminary versions, item review by experts and parents, and a qualitative interview study (27) in a sample of [institution name deleted to maintain integrity of review process] pediatric/medicine-pediatric residents. The final version of the RPQ was then administered to residents at [institution name deleted to maintain integrity of review process] and 14 other sites.

### Questionnaire Measure Components

The RPQ items are grouped in five sections: Demographics, Parenting Advice in Clinic, Perception of Upbringing, Confidence in Providing Parenting Advice, and Parenting Resources. The RPQ can be reviewed in full in Supplementary Image 1.

#### Demographics

Factors that could potentially impact how residents were parented and advice they give were obtained. These included resident age, gender, ethnicity, acculturation (i.e., number of generations their family were in the U.S.), marital status, number of children living in their home, resident level, and future plans. Program and geographic region were also recorded.

#### Parenting Advice in Clinic (PAC)

Ten multiple-choice questions each presented a scenario parents commonly ask residents, and response options were based on Baumrind's categories (17). The Parenting Style Inventory (PSI) was adapted from Walker et al. (unpublished)^1^ as no known published measures assess style of parenting advice provided by a clinician. The PSI was validated with 290 college students and presents 25 multiple-choice questions of common parenting/discipline with 3 response options based on Baumrind's traditional categories and a fourth response representing an action consistent with child maltreatment. Items from the PSI were adapted for the current study. New items were created in order to make questions more relevant to situations of pediatric residents offering current-day parenting advice and to ensure a variety of pediatric age ranges were represented. Participants were categorized based upon their highest frequency of responses consistent with one of the parenting categories (e.g., authoritative, authoritarian, permissive). To achieve relative face validity and because the pilot study yielded an authoritative categorization for all participants, this section was further modified. These modifications were based upon close item review and comparison by a panel of psychologists, general pediatricians, developmental-behavioral pediatricians, and parents and data obtained during the qualitative interview study (27). The questionnaire was then re-piloted with modifications yielding increased variability of categorized responses. Furthermore, data obtained during the interview study was reviewed in-depth by the PI, qualitative advisor, a graduate assistant, and members of the panel and used in restructuring questions and answer options in the PAC section so that information was more representative of what current residents are likely to practice and so that certain answers do not appear more correct than others. Table 1 above illustrates an example of an original question the PSI and the final adapted version presented in the RPQ PAC Section.

**Table 1:** Sample adaptation of parenting style inventory item for inclusion in final version of resident parenting questionnaire parenting advice in clinic section.

Original Parenting Style Inventory Item	You wake up screaming from a nightmare. Your parent… a) tells you to be quiet and to go back to sleep. (*authoritarian*)^a^ b) talks to you about the dream and offers you comfort. (*authoritative*) c) hit you for being loud. (*maltreatment*) d) lets you sleep with him/her. (*permissive*)
Adapted Resident Parenting Questionnaire Item	A 4-years-old child frequently wakes up during the night and comes to his parents' room. His parents would like advice on getting him to sleep in his own bed all night. You initially recommend that his parents: a) Talk about his fears/worries (e.g., scared of the dark) and take him back to his bed if he appears settled. (*permissive*) b) Discuss the reason why he is coming to their room each night, then calmly and consistently put him back in his bed. (*authoritative*) c) Explain the rule that he is to stay in his room at night and take him back to his bed with little interaction. (*authoritarian*)

a *Categories are included here for clarification purposes only; they were not included on the actual questionnaires*.

#### Perception of Upbringing

Because no measure of physician attitudes toward parenting exists, the well-validated Parental Authority Questionnaire (PAQ) (28) was used to assess residents' perception of the parenting they received as children. The PAQ includes 30 Likert-scale items that categorize respondents' parents by Baumrind's parenting styles (10 items each for authoritative, permissive, and authoritarian) (17). Normative, reliability, and validity testing on the PAQ has previously been deemed acceptable in high school and college students (28). Parental Authority Questionnaire (PAQ) responses were used to group residents into the three parenting style categories based on their upbringing. This categorization was achieved by computing the mean response to questions representing each category and assigning participants to the category with the largest mean response.

#### Confidence in Providing Parenting Advice

Confidence in providing parenting advice was assessed in 6 different pediatric age ranges (Infant, Toddler, Preschool, Elementary, Pre-Teen, Adolescent) on a Likert scale with options ranging from Not Very Confident (1) to Very Confident (5).

#### Parenting Resources

Residents were also asked to select various resources from a list (e.g., attending physicians' advice, books, their own parents/growing up) upon which they rely in providing parenting advice in clinic. An open response option was also included to capture resources not listed.

### Survey Data Collection

The RPQ was administered electronically via SurveyMonkey®. Email invitations including a questionnaire overview and link were sent to all study population members with follow-up invitations sent up to five times to those who had not yet responded. Investigators assigned each returned response a research identification number to increase confidentiality. Informed consent was implicit with survey completion as the survey stated participation was completely voluntary and residents could opt to leave a question blank if uncomfortable answering it. Electronic gift cards ($5 value) were offered as compensation for time spent completing surveys.

### Analysis

Based upon pilot questionnaire data, a chi-square power analysis predicted that a sample size of 191 residents would have 80% power to detect a moderate effect size (ω = 0.25), as such, indicating a significant difference among the number of combinations of residents with each type of parenting advice (three PAC levels) and each type of perceived upbringing (three PAQ levels).

Descriptive statistics (frequency/percent) were computed for program, region, age, gender, race, acculturation, marital status, number of children at home, resident level, and future plans. Survey responses to questions about perceived upbringing were classified as permissive, authoritarian, or authoritative by averaging the scores of the three types of PAQ questions, and classifying participants to the group with the highest average value. Similarly, residents were classified by the type of advice they indicated giving in clinic in the PAC section as permissive, authoritarian, or authoritative. In anticipation of having some potentially small cell counts, participants with equal predominance [e.g., ties among 2–3 categories (*n* = 25)] were examined. This allowed for analyses to be conducted with new categories for the ties and with removal of ties.

Associations between PAC category and the categorical variables program, region, resident age groups, gender, race, acculturation, marital status, number of children at home, resident level, and future plans were explored using Chi-square tests or Fisher's exact test as appropriate (i.e., when expected cell counts were small enough so as to violage the assumptions of the Chi-square test). Bowker's test of symmetry was used to determine if and how PAQ was associated with PAC. This test for symmetry was done with all participants both creating new classes for the ties and also by removing those subjects with ties. Simple and multivariable logistic regression models were used to test associations between clinic advice and perceived upbringing while controlling for other covariates.

Overall confidence ratings for giving advice for children of various age ranges were tabulated, and comparisons between levels were made using chi-square test/Fisher's exact tests. Iteratively reweighted least squares regression (i.e., robust regression) tested the effect of child age on residents' confidence giving advice while controlling for other covariates. Resident confidence levels were also treated as a continuous variable, tested for normality using the Shapiro-Wilk test, with median (25th, 75th) or mean (SD) reported.

Resident resource use (i.e., never/rarely, occasionally, sometimes, or often) results were tabulated, and frequencies with corresponding percentages were reported. Bowker's test of symmetry was used to compare similarity of resource use between pairs of the various resources available.

## Results

### Demographic Data

Three-hundred and twenty-seven residents attempted the survey with 301 participants providing overall adequate demographic response sets. Table 2 lists demographic information, and Table 3 outlines the geographic breakdown of resident participation among the 15 U.S. pediatric residency programs. The average response rate/program was 33.1% with an overall survey completion rate of 27.9% and uniform representation across U.S. regions. Compared to Accreditation Council for Graduate Medical Education national demographic information available for U.S. pediatric and medicine-pediatric residency programs at that time (26), participants were similar in age and gender make-up, but the sample appears to have a predominance of non-Hispanic Caucasian participants. Comparison of study participant demographics to program-level information (e.g., age range, gender, race/ethnicity) for all participating programs has not been included as collection methods and level of response varied among programs.

**Table 2:** Resident parenting questionnaire participant sample demographics (*N* = 301)^a^.

**Characteristic**	***n* (%)**
**AGE**	
25–29	202 (67.8%)
30–34	87 (29.2%)
35–39	6 (2.0%)
40–44	1 (0.3%)
45–49	1 (0.3%)
50–55	1 (0.3%)
**GENDER**	
Female	235 (79.4%)
Male	61 (20.6%)
**RACE/ETHNICITY**	
White/Caucasian	214 (71.3%)
Asian	49 (16.3%)
Hispanic/Latino	14 (4.7%)
Black/African American	8 (2.7%)
American Indian	2 (0.7%)
Native Hawaiian/Other Pacific Islander	1 (0.3%)
Other	12 (4.0%)
**GENERATION IN U.S**.	
>2nd	192 (64.0%)
2nd	26 (8.7%)
1st	50 (16.7%)
Immigrant	21 (7.0%)
Student Visa	10 (3.3%)
Green Card	1 (0.3%)
**MARITAL STATUS**	
Married	168 (56.2%)
Never Been Married	123 (41.1%)
Domestic Partnership	5 (1.7%)
Divorced/Separated	3 (1.0%)
**#CHILDREN**	
0	232 (77.3%)
1	40 (13.3%)
2	27 (9%)
3	1 (0.3%)
**RESIDENCY PROGRAM**	
General Pediatrics	262 (87.0%)
Internal Medicine/Pediatrics	27 (9.0%)
Other	12 (4.0%)
**RESIDENT LEVEL (i.e., Year in Residency)**	
1st	92 (30.6%)
2nd	102 (33.9%)
3rd	91 (30.2%)
4th	13 (4.3%)
5th	2 (0.7%)
**FUTURE PLANS**	
Subspecialty	130 (43.5%)
Primary Care	114 (38.1%)
Hospitalist	38 (12.7%)
Other	17 (5.7%)

a *Unaccounted responses in each section above represent missing/incomplete responses within each section*.

**Table 3:** Resident parenting questionnaire participant geographic breakdown.

**Program region**	**#of Programs**	**Frequency (% total participants)**
Northeast	4	64 (21.3%)
Midwest	3	81 (26.9%)
South	4	70 (23.3%)
West	4	86 (28.6%)
Total	15^a^	301 (27.9%)^b^/Average response rate/program = 33.1%

a *Out of 199 programs*.

b *Total response rate = 301 adequately completed surveys out of 1079 total possible participants*.

### Residents' Upbringing and Parenting Advice They Give

Of the 301 participants with overall adequate response sets, 290 provided complete PAQ and PAC sections. Of these 290, 196 (68%) indicated authoritative upbringing, 85 (29%) indicated authoritarian upbringing, and 4 (1.4%) indicated permissive upbringing. Five participants had ties among PAQ categories [3 (1%) authoritarian/authoritative and 2 (0.7%) authoritarian/authoritative/permissive]. Regarding parenting advice type preferred based on PAC results, 241 (83%) participants indicated giving authoritative advice, 7 (2.4%) indicated giving authoritarian advice, and 21 (7.2%) indicated giving permissive advice. Twenty-one participants had ties among PAC categories [4 (1.4%) authoritarian/authoritative and 17 (5.9%) authoritative/permissive]. Regardless of perceived upbringing, the majority of residents reported giving authoritative advice (90% when those with tie categories were excluded). However, based on Bowker's test of symmetry (see Table 4), there is significant evidence to suggest that residents' parenting advice does differ based on how they were parented (*p* < 0.001). Specifically, those who perceived their upbringing as authoritative were more likely to give authoritative parenting advice in clinic (85%) than authoritarian or permissive advice. However, participants who perceived their upbringing as authoritarian or permissive tended to give parenting advice that contrasted with how they were raised. Specifically, those perceiving an authoritarian upbringing were more likely to give authoritative (80%) or permissive (8.2%) parenting advice (2.5% reported giving authoritarian advice). Additionally, those perceiving a permissive upbringing were more likely to give authoritative (75%) or authoritarian (25%) parenting advice (none reported giving permissive advice). These results remained consistent even when analyses removed those with category ties.

**Table 4:** Analysis of how residents were parented in childhood and the type of parenting advice they give in clinic based on resident parenting questionnaire sample (*N* = 290)^a^.

		**Type of advice residents give in clinic**					
		**Authoritarian**	**Authoritative**	**Permissive**	**A/A**	**A/P**	***p*-value**
How Residents Were Parented	Authoritarian	2 (2.5%)	68 (80%)	7 (8.2%)	3 (3.5%)	5 (5.9%)	<0.001^b^
	Authoritative	4 (2%)	167 (85%)	13 (6.6%)	0 (0%)	12 (6.1%)	
	Permissive	1 (25%)	3 (75%)	0 (0%)	0 (0%)	0 (0%)	
	A/A	0 (1%)	2 (66.7%)	0 (0%)	1 (33.3%)	0 (0%)	
	A/A/P	0 (0%)	1 (50%)	1 (50%)	0 (0%)	0 (0%)	

*A/A, Authoritarian/Authoritative; A/P, Authoritative/Permissive; A/A/P, Authoritarian/Authoritative/Permissive*.

a *290 participants had complete response sets for the Parental Authority Questionnaire and Parenting Advice in Clinic sections*.

b *Results remained significant when all participants with category ties removed*.

### Demographic Covariates and Parenting Advice in Clinic

Chi-square/Fisher's exact tests indicated that, overall, there were significant or marginally significant associations between parenting advice and resident race (*p* = 0.0385), acculturation (i.e., number of generations in U.S.; *p* = 0.0630), and future plans (*p* = 0.0449). When analyzing pairwise comparisons of advice type for these variables, no specific race associations were found. However, residents whose families had been in the U.S. less than two generations were more likely to give permissive advice while those whose families had been in the US two or more generations were more likely to give authoritative advice (*p* = 0.0191). Additionally, residents going into subspecialties were more likely to give permissive advice while those going into primary care were more likely to give authoritative advice (*p* = 0.0233).

Parenting advice was not significantly related to how residents were parented while controlling for future plans and resident level based upon analyses with simple (*p* = 0.5388) and multivariable logistic regression (*p* = 0.1877). Due to high correlation between race and acculturation as well as the significant imbalance of clinic advice levels between authoritative and the other advice categories, no other variables could be adequately included in these models.

### Confidence in Giving Parenting Advice

Table 5 lists residents' median and mean reported confidence levels for all pediatric age ranges. Participants' median confidence in giving parenting advice decreased as patient age increased. Residents were confident in giving parenting advice in infant, toddler, and preschool age ranges but reported more ambivalence and lack of confidence in giving parenting advice in older age ranges. This relationship of confidence in giving parenting advice generally decreasing as patient age increases was also seen when using Chi-square/Fisher's exact tests.

**Table 5:** Resident parenting questionnaire self-reported median and mean confidence in giving parenting advice by pediatric age group.

	**Confidence level^a^**	
**Age group**	**Median (25th−75th%)^b^**	**Mean (SD)**
Infant Confidence	4.00^a^ (4.00, 4.00)	3.83 (0.78)
Toddler Confidence	4.00^a^ (3.00, 4.00)	3.60 (0.76)
Preschool Confidence	4.00^b^ (3.00, 4.00)	3.44 (0.74)
Elementary Confidence	3.00^b^ (3.00, 4.00)	3.39 (0.72)
Pre-Teen Confidence	3.00^c^ (3.00, 4.00)	3.22 (0.83)
Adolescent Confidence	3.00^d^ (2.00, 4.00)	3.17 (0.93)

a *Confidence level responses were assessed for normality using the Shapiro-Wilk test and found to not be*.

*normally distributed. Therefore, medians are more appropriate for comparisons*.

b *Medians with the same superscript are NOT significantly different*.

Robust regression analyses indicated that residents' perception of how they were parented was not associated with their confidence in giving parenting advice in any age range. Table 6 lists all significant associations (*p* < 0.050) found upon analysis of the study covariates and participants' confidence in giving parenting advice in various pediatric age ranges. Resident level was positively associated with level of confidence residents have giving parenting advice in pediatric age ranges from toddler to adolescent. Number of children living in the home was positively associated with confidence giving advice in younger pediatric age ranges. Residents' future plans had associations with confidence giving advice in older pediatric age ranges.

**Table 6:** Associations between resident parenting questionnaire self-reported confidence in giving parenting advice and study covariates^a^.

**Covariate**	**Estimate**	***p*-value**
**TODDLER**		
Years in Residency	0.06755	0.046
Number of Children in Home	0.16833	0.0004
**PRESCHOOL**		
Year in Residency	0.11924	0.008
Number of Children in Home	0.16573	0.009
**ELEMENTARY SCHOOL**		
Year in Residency	0.17500	<0.001
Number of Children in Home	0.10091	0.102
**PRE-TEEN**		
Year in Residency	0.21669	<0.001
Future Plans (ref is other)	0.016	
Hospitalist	−0.06462	
Primary Care	−0.29378	
Subspecialty Fellowship	0.01031^b^	
**ADOLESCENT**		
Year in Residency	0.15268	0.012
Future Plans (ref is other)	0.021	
Hospitalist	0.05822	
Primary Care	−0.22224	
Subspecialty Fellowship	0.10886^b^	

a *There were no other significant associations between covariates and confidence in giving parenting advice in any pediatric age range; of note, Infant was the only age range with no significant associations with confidence identified*.

b *For respondents with future plans of a “Subspecialty fellow,” the confidence scores were significantly higher than for those with future plans of “Primary Care” (p = 0.0047 for Pre-Teen, p = 0.0067 for Adolescent)*.

### Use of Parenting Resources

Residents most frequently reported relying upon attending physician advice when compared to other resources (Bowker's *p* < 0.050 for all comparisons). They also reported sometimes referring to their own parents, family, and friends, books, and AAP/other guidelines to generate their parenting advice. Table 7 lists which parenting resources were cited as being used most and least often by the residents queried.

**Table 7:** Resident parenting questionnaire self-reported frequency of resources used in generating parenting advice.

**Most frequently used resources** (% that report **Often** using this resource)	
**Resource**	**Frequency (%)**
Attending Physician	194 (71.3%)
Advice from own parents/family/friends	61 (22.8%)
AAP Guidelines	56 (20.7%)
Books/Reading Material	52 (19.2%)
Didactic Presentations	34 (12.6%)
**Least frequently used resources** (% that report **Never/Rarely** using this resource)	
**Resource**	**Frequency (%)**
Other Resources	169 (92.86%)
Personal Experience	201 (75.00%)^a^
Formal Skills Training	170 (63.67%)
Training/Lectures prior to Residency	120 (44.61%)
Website	91 (33.83%)
Recall from Childhood	54 (20.15%)

a *77% of participants reported that they have no children living in their home*.

## Discussion

Relatively little has been known about the resources pediatric residents use to generate their parenting advice in clinic, and given that parental behavior is known to be passed through generations (16), understanding whether residents base their guidance on their upbringing is essential. Our study indicates that residents raised in an authoritative manner reported that they largely give authoritative advice in clinic, but most residents raised in the other styles also reported favoring authoritative advice. Of interest in both this study and the associated interview study (27), residents raised in authoritarian or permissive manners rarely indicated giving that same type of advice, and results suggest they sometimes overcompensate to ensure their advice contrasts their own upbringing. Most pediatric residents appear to be aware of appropriate authoritative parenting advice regardless of upbringing, especially as they progress through residency, suggesting that current training in parenting advice may be adequate in general for the majority of residents. However, there may be subtle deviations from authoritative advice for residents not raised authoritatively or for those preferring to go into certain subspecialties as opposed to primary care. Therefore, opportunities to reflect upon their own upbringing could help some residents unpack any hidden biases in their parenting advice.

Our study's analyses suggest that residents' race, generational U.S. background, future plans, and resident level are all potentially associated with their parenting advice. In regard to a relationship between residents' race/ethnicity and preferred type of parenting advice, it is difficult to say whether there is a definite, specific relationship based upon this study. Although there was an overall significant association, no specific relationship could be determined likely due to a sample size decrease once comparing two types of advice directly with the third category eliminated. It is possible with further investigation of a larger sample size, a meaningful variation in advice preference by race/ethnicity groups could be elicited. Given that research has indicated that cultural differences exist in parenting (29–31), subtle variations in parenting advice among residents of different race/ethnicity background could be possible.

A growing body of literature has also indicated that developmental and behavioral outcomes are strongly associated with a family's degree of acculturation to U.S. customs and practices (32–34). This suggests that, like race/ethnicity variations, approaches to parenting could vary by number of generations one's family has been in the U.S. Our study suggests that this was possibly present among residents surveyed as those whose families have been in the U.S. <2 generations were found to give more permissive parenting advice while those whose families have lived in the U.S. longer report giving more authoritative advice. Potential mechanisms underlying this association are likely complex, and it is possible that social/cultural factors closely linked or interacting with acculturation were not adequately represented in our study.

Residents appear to be more confident in giving parenting advice for younger children, particularly infants, compared with older children and adolescents. This likely reflects that they are exposed to a larger volume of infant and toddler well child examinations during which general parenting advice typically plays a fairly prominent role. As older children and adolescents are developing more autonomy and sometimes experiencing more conflict with parents, more challenges are posed in implementing physician guidance and effective communication related to parenting during visits with the older pediatric patients. Residents are typically closer in age to adolescents and therefore may not have the perspective of time to assess their own experiences in older children. Furthermore, their faculty may be less skilled in providing parenting advice for adolescents. Confidence in these pre-teen/adolescent visits may improve as Adolescent Medicine rotations begin to play a more prominent role in pediatric residency programs.

In general, confidence related to provision of parenting advice appears to improve for all pediatric patients as residents advance through residency. However, even residents in their final year of training still do not largely report that they are very confident in providing parenting advice suggesting there still may be room for improving their sense of efficacy around advising on basic parenting topics. Residents with more children at home also reported higher levels of confidence giving parenting advice, which was also a key finding in the associated qualitative study (27). This effect was only significant in younger patient age ranges though, likely due to the fact that most residents with children do not yet have older children themselves.

Residents in our study reported being far more likely to consult their attending physicians for guidance on provision of parenting advice than to access AAP guidelines or other evidence-based resources. They also are not largely being exposed to formal training or didactic sessions related to parenting strategies. Although the content of participants' advice, as reported, appears to be fairly appropriate, the extent of training that pediatric residency faculty physicians have in providing evidence-based parenting advice themselves is unknown.

### Limitations

Because no existing measures were identified that directly related to pediatric residents' upbringing and parenting advice, a new instrument was developed. Although standard psychometric validation procedures were not pursued, relative face validity was achieved by having items in the Parenting Advice in Clinic section rigorously and iteratively reviewed by a panel of expert psychologists, general pediatricians, developmental-behavioral pediatricians, qualitative interviewers, and parents with a goal of having items in this section represent advice they may give in clinic yet not having any response seem more “correct” than another. The RPQ may not have accurately portrayed advice residents truly give, particularly given that we were unable to assess residents' advice-giving through direct observation. The survey was also subject to recall and social desirability response bias. It is possible that residents may not be fully aware of underlying biases that come across in their actual clinic visits but were more aware of answers that appeared to be correct in survey/test format despite efforts to reduce this effect. Additionally, the RPQ responses in our study were potentially susceptible to item response fatigue, particularly the PAQ items which were in the final section. During the pilot phase of this study, these items were intentionally placed later due to concern that the more sensitive questions about participants' upbringing may have been difficult to immediately answer. This, however, may have resulted in more items going unanswered in this section. The authors also acknowledge that the wording of the questions in the RPQ Parenting Resources section missed an opportunity to ask residents to specify if they were exposed to any particular evidence-based parenting program, rather than just an inquiry of the format or methods that these skills are presented to them. Finally, our response rate was slightly lower than general survey response rates, which may have increased the homogeneity of responses; therefore, caution should be used when generalizing the findings. However, it was fairly comparable to several other national, web-based medical resident and pediatrician survey studies (35–40). While it did not likely represent experiences in all pediatric residency programs, the sample surveyed was large and fairly nationally representative.

### Implications

Results of this study suggest that developing formalized pediatric resident parenting curriculum would likely provide residents with more structured understanding of best practices and more evidence-based resources in parenting recommendations. Having access to these tools could improve confidence in parenting advice provision, and incorporating curriculum components within existing Adolescent Medicine rotations could be especially helpful for improving confidence around parenting advice to pre-teens/adolescents. Knowing that residents without children report relatively lower confidence, programs could also consider including more observation opportunities outside the clinic setting (e.g., daycares, preschools) or sessions to hear perspectives of parents who are not themselves physicians. As previously noted, residents may benefit from exercises allowing them to reflect upon their upbringing, particularly if raised in authoritarian or permissive styles. Including initial training sessions for faculty attending physicians and involving senior residents in curricular training/didactic session development would be key to successful, long-lasting, and meaningful curriculum implementation. Finally, although more expensive and logistically challenging, to truly understand and assess what types of advice residents give, incorporation of direct standardized or actual clinical observations into a resident parenting curriculum is recommended. This is currently being done at Indiana University (13) and, if more widely implemented, would allow faculty at other institutions to provide direct feedback on parenting advice content and delivery. Implementing measures to strengthen pediatric residents' parenting advice has the potential to improve physician-family relationships and outcomes related to child behavioral and family well-being.

## Data Availability Statement

The raw data supporting the conclusions of this manuscript will be made available by the authors, without undue reservation, to any qualified researcher. It is not publicly available at this time as this was not agreed upon during the resident participant consent or Institutional Review Board approval processes.

## Ethics Statement

This study was carried out in accordance with the Declaration of Helsinki and recommendations of University of Oklahoma Health Sciences Center Institutional Review Board. The protocol was approved by the University of Oklahoma Health Sciences Center Institutional Review Board. Informed consent was implicit with survey completion as the survey stated participation was completely voluntary and residents could opt to leave a question blank if uncomfortable answering it.

## Author Contributions

AB contributed substantially to the study conception and design, data acquisition and interpretation, and drafting, critical revision, and final approval of the manuscript. PS contributed to the study design, data acquisition, analysis, and interpretation, and drafting, critical revision, and final approval of the manuscript. MA contributed to the study design, data analysis and interpretation, and drafting, critical revision, and final approval of the manuscript. MW contributed to the study conception and design, data interpretation, and critical revision and final approval of the manuscript. AB, PS, MA, and MW all agree to be accountable for all aspects of the work in ensuring that questions related to the accuracy or integrity of any part of the work are appropriately investigated and resolved.

### Conflict of Interest Statement

The authors declare that the research was conducted in the absence of any commercial or financial relationships that could be construed as a potential conflict of interest.

## Footnotes

^1^Katzung VM, Thigpen SM, Jacobs JT, Walker CE. Construction and Validation of the Parenting Style Inventory. In: *Paper Presented at the Annual Meeting of the Oklahoma Psychological Society and the Oklahoma Psychological Association Spring Meeting*. Oklahoma City, OK (1991).

## Abbreviations

AAP: American Academy of Pediatrics
PAC: Parenting Advice in Clinic
PAQ: Parental Authority Questionnaire
PSI: Parenting Style Inventory
U.S.: United States.
