# Supplementary material for: The Relationship Between Pediatric Residents' Experiences Being Parented and Their Provision of Parenting Advice
Source: Front Pediatr. 2018 Dec 12;6:395. doi: 10.3389/fped.2018.00395 (PMC6299100; doi:10.3389/fped.2018.00395)
Supplement: Supplementary file 1 [file Image_1.PDF]

Resident Parenting Questionnaire

DEMOGRAPHICS

1. Please select your age category.

- 20-24
- 25-29
- 30-34
- 35-39
- 40-44
- 45-49
- 50-55

2. Gender

- Male
- Female

3. Please select the race/ethnicity category with which you most identify. Select all that apply.

- American Indian/Alaskan Native
- Hispanic/Latino
- Asian
- Native Hawaiian/Other Pacific Islander
- Black/African-American
- White
- Other (please specify)

4. Which generation American below best describes you?

- Here on student visa
- Immigrant
- 1st-generation American
- 2nd-generation American
- >2nd-generation American

Optional Comment:

5. Please describe your marital status.

- Never Been Married
- Married
- Domestic Partnership
- Divorced/Separated
- Widowed

6. How many children live in your home?

- 0
- 1
- 2
- 3
- >3

7. If you have children living in your home, what is the age of the oldest child? If <2 years old, please list in months.

8. Please list your residency program type.

- General Pediatrics
- Internal Medicine-Pediatrics
- Community Pediatrics
- Family Medicine
- Other (Please specify)

Human Research Participant Protection The University of Oklahoma IRB APPROVED AAHRPP
IRB NUMBER: 1477
IRB APPROVAL DATE: 11/24/2014

9. Please list your Resident Post-Graduate Year (PGY) status.

- PGY-1
- PGY-2
- PGY-3
- PGY-4
- Other (please specify)

10. Which of the following best describes your plans following completion of residency?

- Primary Care
- Hospitalist
- Subspecialty Fellowship
- Other (please specify)

11. If you are planning on doing a fellowship, which subspecialty?

Human Research Participant Protection
The University of Oklahoma
IRB APPROVED
AAHRPP
IRB NUMBER: 1477
IRB APPROVAL DATE: 11/24/2014

Resident Parenting Questionnaire

PARENTING ADVICE IN CLINIC

Please base the following answers upon ADVICE YOU WOULD MOST LIKELY GIVE to a parent in your clinic regarding PARENTING/DISCIPLINE in the following scenarios. Do not worry about selecting a "correct answer," as there isn't a single correct response to these questions.

• Select the advice that you believe to be the MOST IMPORTANT INITIAL RECOMMENDATION to the parents.

• Assume recommendations that include referrals (ie counseling, therapy, specialists), medication therapy, or diagnostic workups are not indicated at this time.

• Please assume all scenarios involve:
- two parents with adequate resources/mid-level income and no significant physical or mental health problems AND
- children that are healthy and typically developing.

1. A 4-year-old child frequently wakes up during the night and comes to his parents' room. His parents would like advice on getting him to sleep in his own bed all night. You initially recommend that his parents:

- ○ Talk about his fears/worries (e.g. scared of the dark) and take him back to his bed if he appears settled.
- ○ Discuss the reason why he is coming to their room each night, then calmly and consistently put him back in his bed.
- ○ Explain the rule that he is to stay in his room at night and take him back to his bed with little interaction.

2. A 6-year-old child with average BMI refuses to eat most foods prepared by her parents for dinner. Her parents would like advice on improving her nutritional intake. You initially recommend that:

- ○ Food battles should be avoided whenever possible to decrease added stress and make mealtime more enjoyable.
- ○ Parents should continue to offer the same selections because she will eventually eat when she is hungry enough.
- ○ The child's preferences should be considered in meal-planning, but less desired items should also be consistently offered.

3. A 3-year-old child is not yet toilet-trained. Half of the time, she seems to understand when she needs to void or stool but becomes very anxious when taken to the bathroom. The other half of the time, she voids or stools in the middle of playing. Her parents would like advice on how to achieve toileting independence. You initially recommend that they:

- ○ Back off a structured toileting routine because it may be increasing stress if she is not developmentally ready for toilet training.
- ○ Initiate a consistent toileting routine at select times each day and provide rewards when the child voids in the toilet.
- ○ Implement consistent toilet practices and remove her from play as she likely will not stop playing to go to the bathroom.

Human Research Participant Protection The University of Oklahoma IRB APPROVED AAHRPP
IRB NUMBER: 1477
IRB APPROVAL DATE: 11/24/2014

4. A 6-month old infant is frequently refusing his naps and is fussy and cries a lot when his parents try to put him in his crib. His parents would like advice on what to do when he won't fall asleep at naptime. You initially recommend that with each crying episode, they assess whether he needs a diaper change, is hungry, or is hurt. If none of these are present, you recommend that they:

- Implement a pre-naptime routine, calmly rubbing his back or walking with him in a stroller to calm him down.
- Consistently place him in his crib calmly saying, "Time for nap," which should eventually establish naps.
- Gently rock and soothe him at naptime, and if he still resists, consider that he may no longer need to nap at that time.

5. A 16-year-old girl has gradually been using her cell phone more and is now staying awake many nights texting and talking to friends a few hours past her 10:00 pm bedtime. Her parents would like advice on getting her to decrease her cell phone use, and you initially recommend that they:

- Restrict her from her cell phone for one week because it is likely distracting her from studying and sleep.
- Set structured limits on her cell phone use prior to bedtime and explain the importance of her getting adequate sleep.
- Engage in negotiations providing her the opportunity to propose cell phone limits to promote her independent decision-making.

6. A parent tells you that their 5-year-old son has taken candy without permission twice now while grocery shopping with the family. You initially recommend that his parents should:

- Provide increased interaction when shopping and place him in time-out immediately in the store if it happens again.
- Require him to return the candy and describe how stealing is not acceptable in their family and is against the law.
- Tell him stealing is wrong and increase attention and treats for good behavior while shopping as he may be feeling ignored.

7. Parents express that their 8-year-old child is not cooperating with them when doing his homework. He completes similar assignments at school without difficulty. You initially advise that they:

- Place him in time-out if he does not remain seated at the table to complete his homework.
- Allow him time to play first because it may decrease his stress and help him better focus on homework.
- Involve him in selecting rewards that he can earn for completing his homework each day.

IRB NUMBER: 1477
IRB APPROVAL DATE: 11/24/2014

8. A 3-year-old child has been throwing tantrums every night when not allowed to stay up past her usual bedtime to watch television shows. You initially recommend that her parents:

- Implement a family rule that the television is off after her bedtime and use a sticker chart to reward times she stays in bed.
- Engage in a fun but calming activity with her before bedtime and allow her to gradually calm down and become sleepy.
- Remind her there is no television use after bedtime and return her to bed whenever she tries to come watch television.

9. Parents decide their family must move to a new town, and their 13-year-old child is very upset. You initially advise that the parents:

- Explain that the family must move and things will most likely be fine after they get to their new town.
- Have a family meeting to discuss the reasons the family must move and how his concerns may be met.
- Buy him a favorite video game or other desired item to ease the difficulty of the family's move.

10. A 7-year-old child's grandparent has recently died, and her parents say she has been acting out more than usual. You initially advise her parents to:

- Maintain their usual school/home activities routine and read books together about losing a grandparent.
- Engage in open discussion of her feelings and relax their usual discipline routine while she appears upset.
- Encourage her to be strong and not feel sorry for herself and continue their usual discipline and activities routine.

Human Research Participant Protection
The University of Oklahoma
IRB APPROVED
AAHRPP
IRB NUMBER: 1477
IRB APPROVAL DATE: 11/24/2014

Resident Parenting Questionnaire

CONFIDENCE IN GIVING PARENTING ADVICE

Please describe your confidence in discussing PARENTING AND DISCIPLINE strategies with parents of your patients in the following age ranges:

1. Infants (0-1 year)

- 1= Not very confident
- 2= Not confident
- 3= Ambivalent
- 4= Confident
- 5= Very confident

2. Toddlers (1-4 years)

- 1= Not very confident
- 2= Not confident
- 3= Ambivalent
- 4= Confident
- 5= Very confident

3. Preschool Aged (4-6 years)

- 1= Not very confident
- 2= Not confident
- 3= Ambivalent
- 4= Confident
- 5= Very confident

4. Elementary School-Aged (6-10 years)

- 1= Not very confident
- 2= Not confident
- 3= Ambivalent
- 4= Confident
- 5= Very confident

5. Pre-Teens (10-13 years)

- 1= Not very confident
- 2= Not confident
- 3= Ambivalent
- 4= Confident
- 5= Very confident

6. Adolescents (13-21 years)

- 1= Not very confident
- 2= Not confident
- 3= Ambivalent
- 4= Confident
- 5= Very confident

Human Research Participant Protection
The University of Oklahoma
IRB APPROVED
AAHRPP
IRB NUMBER: 1477
IRB APPROVAL DATE: 11/24/2014

Resident Parenting Questionnaire

PARENTING RESOURCES

1. The question below has two parts:

A. Please mark the components that are included as part of the parenting and discipline training in your program.

B. Please mark which components you have received to date as part of your training.

	Didactic Presentations	Formal Skill Training	Assigned Reading Material	Clinical Supervision	None of these are included
A. Offered in program	☐	☐	☐	☐	☐
B. Received to date	☐	☐	☐	☐	☐

2. Please list any other components not mentioned above that are included as a part of the parenting and discipline training in your program.

- ○ No other components are offered as a part of my program
- ○ Other (please specify)

Human Research Participant Protection The University of Oklahoma IRB APPROVED AAHRPP
IRB NUMBER: 1477
IRB APPROVAL DATE: 11/24/2014

3. Please describe how frequently you rely upon the following resources in giving parenting and discipline advice.

	Never/Rarely	Occasionally	Sometimes	Often
AAP/Other Guidelines	○	○	○	○
Advice from Own Parents or Family Members/Friends that are Parents	○	○	○	○
Attending Physician Advice	○	○	○	○
Books/Reading Material	○	○	○	○
Didactic Presentations	○	○	○	○
Formal Skill Training	○	○	○	○
Personal Experience as a Parent	○	○	○	○
Recall from Childhood Experience	○	○	○	○
Trainings/Lectures Prior to Residency	○	○	○	○
Web Searches/Websites	○	○	○	○
Other	○	○	○	○

If you selected Other, please explain.

Human Research Participant Protection The University of Oklahoma IRB APPROVED AAHRPP
IRB NUMBER: 1477
IRB APPROVAL DATE: 11/24/2014

Resident Parenting Questionnaire

WHO PARENTED YOU?

Please base the answers in this section on YOUR CHILDHOOD (birth through high school).

1. Please describe the type(s) of caregivers that lived in the home with you when you were growing up. You may choose more than one.

- [ ] biological mother
- [ ] grandmother
- [ ] adoptive mother
- [ ] biological father
- [ ] grandfather
- [ ] adoptive father
- [ ] step-mother
- [ ] other relative
- [ ] other (explain below)
- [ ] step-father
- [ ] foster parent(s)

Please clarify if necessary (OPTIONAL):

2. Of those you chose in #1, who was primarily responsible for your discipline when you were growing up? You may choose more than one.
***Note: PLEASE REMEMBER YOUR ANSWER as you will be asked to think of this person/these people in questions on the following page.**

- [ ] biological mother
- [ ] grandmother
- [ ] adoptive mother
- [ ] biological father
- [ ] grandfather
- [ ] adoptive father
- [ ] step-mother
- [ ] other relative
- [ ] other (explain below)
- [ ] step-father
- [ ] foster parent(s)

Please clarify if necessary (OPTIONAL):

3. If there was more than 1 person responsible for your discipline, how often did they agree about parenting decisions?

- ( ) Always
- ( ) Almost always
- ( ) Half the time
- ( ) Almost never
- ( ) Never

4. (Optional) Please explain any caretaker circumstances you had that you feel need further clarification (e.g. change in custody, living, or social situation).

Human Research Participant Protection The University of Oklahoma IRB APPROVED AAHRPP
IRB NUMBER: 1477
IRB APPROVAL DATE: 11/24/2014

Resident Parenting Questionnaire

PARENTAL AUTHORITY QUESTIONNAIRE

In answering the following 30 questions, where it says "PARENT," please refer to your perceptions of how the CAREGIVER(S) you chose on the previous page disciplined you.

INSTRUCTIONS: For each of the following statements, choose the number (from 1 = strongly disagree to 5 = strongly agree) that best describes how the statement applies to you and your parent.

As you read each statement, think about how it applies to you and your parent during your years of growing up at home.

There are no right or wrong answers, so you do not have to spend a lot of time on any one item.

We are looking for your overall impression regarding each statement.

1 = Strongly DISAGREE, 2 = DISAGREE, 3 = Neither DISAGREE nor AGREE, 4 = AGREE, 5 = Strongly AGREE

1. Q1

	1	2	3	4	5
While I was growing up my parent felt that in a well-run home the children should have their way in the family as often as the parents do.	○	○	○	○	○

2. Q2

	1	2	3	4	5
Even if his/her children didn’t agree with him/her, my parent felt that it was for our own good if we were forced to conform to what he/she thought was right.	○	○	○	○	○

3. Q3

	1	2	3	4	5
Whenever my parent told me to do something as I was growing up, he/she expected me to do it immediately without asking any questions.	○	○	○	○	○

4. Q4

	1	2	3	4	5
As I was growing up, once family policy had been established, my parent discussed the reasoning behind the policy with the children in the family.	○	○	○	○	○

5. Q5

	1	2	3	4	5
My parent has always encouraged verbal give-and-take whenever I have felt that family rules and restrictions were unreasonable.	○	○	○	○	○

6. Q6

	1	2	3	4	5
My parent has always felt that what his/her children need is to be free to make up their own minds and to do what they want to do, even if this does not agree with what their parents might want.	○	○	○	○	○

7. Q7

	1	2	3	4	5
As I was growing up my parent did not allow me to question any decision he/she had made.	○	○	○	○	○

8. Q8

	1	2	3	4	5
As I was growing up my parent directed the activities and decisions of the children in the family through reasoning and discipline.	◯	◯	◯	◯	◯

9. Q9

	1	2	3	4	5
My parent has always felt that more force should be used by parents in order to get their children to behave the way they are supposed to.	◯	◯	◯	◯	◯

10. Q10

	1	2	3	4	5
As I was growing up my parent did not feel that I needed to obey rules and regulations of behavior simply because someone in authority had established them.	◯	◯	◯	◯	◯

11. Q11

	1	2	3	4	5
As I was growing up I knew what my parent expected of me in my family, but I also felt free to discuss those expectations with my parent when I felt that they were unreasonable.	◯	◯	◯	◯	◯

12. Q12

	1	2	3	4	5
My parent felt that wise parents should teach their children early just who is boss in the family.	◯	◯	◯	◯	◯

13. Q13

	1	2	3	4	5
As I was growing up, my parent seldom gave me expectations and guidelines for my behavior.	◯	◯	◯	◯	◯

14. Q14

	1	2	3	4	5
Most of the time as I was growing up my parent did what the children in the family wanted when making family decisions.	◯	◯	◯	◯	◯

15. Q15

	1	2	3	4	5
As the children in my family were growing up, my parent consistently gave us direction and guidance in rational and objective ways.	◯	◯	◯	◯	◯

16. Q16

	1	2	3	4	5
As I was growing up my parent would get very upset if I tried to disagree with him/her.	◯	◯	◯	◯	◯

17. Q17

	1	2	3	4	5
My parent felt that most problems in society would be solved if parents would not restrict their children's activities, decisions, and desires as they are growing up.	◯	◯	◯	◯	◯

Human Research Participant Protection
The University of Oklahoma
IRB APPROVED
AAHRPP
IRB NUMBER: 1477
IRB APPROVAL DATE: 11/24/2014

18. Q18

	1	2	3	4	5
As I was growing up my parent let me know what behavior he/she expected of me, and if I didn't meet those expectations, he/she punished me.	◯	◯	◯	◯	◯

19. Q19

	1	2	3	4	5
As I was growing up my parent allowed me to decide most things for myself without a lot of direction from him/her.	◯	◯	◯	◯	◯

20. Q20

	1	2	3	4	5
As I was growing up my parent took the children's opinions into consideration when making family decisions, but he/she would not decide on something simply because the children wanted it.	◯	◯	◯	◯	◯

21. Q21

	1	2	3	4	5
My parent did not view him/herself as responsible for directing and guiding my behavior as I was growing up.	◯	◯	◯	◯	◯

22. Q22

	1	2	3	4	5
My parent had clear standards of behavior for the children in our home as I was growing up, but he/she was willing to adjust those standards to the needs of each of the individual children in the family.	◯	◯	◯	◯	◯

23. Q23

	1	2	3	4	5
My parent gave me direction for my behavior and activities as I was growing up and he/she expected me to follow his/her direction, but he/she was always willing to listen to my concerns and to discuss that direction with me.	◯	◯	◯	◯	◯

24. Q24

	1	2	3	4	5
As I was growing up my parent allowed me to form my own point of view on family matters and he/she generally allowed me to decide for myself what I was going to do.	◯	◯	◯	◯	◯

25. Q25

	1	2	3	4	5
As I was growing up my parent always felt that most problems in society would be solved if we could get parents to strictly and forcibly deal with their children when they don't do what they are supposed to as they are growing up.	◯	◯	◯	◯	◯

26. Q26

	1	2	3	4	5
As I was growing up my parent often told me exactly what he/she wanted me to do and how he/she expected me to do it.	◯	◯	◯	◯	◯

27. Q27

	1	2	3	4	5
As I was growing up my parent gave me clear direction for my behaviors and activities, but he/she was also understanding when I disagreed with him/her.	◯	◯	◯	◯	◯

Human Research Participant Protection
The University of Oklahoma
IRB APPROVED
AAHRPP
IRB NUMBER: 1477
IRB APPROVAL DATE: 11/24/2014

Resident Parenting Questionnaire

28. Q28

	1	2	3	4	5
As I was growing up my parent did not direct the behaviors, activities, and desires of the children in the family.	○	○	○	○	○

29. Q29

	1	2	3	4	5
As I was growing up I knew what my parent expected of me in the family and he/she insisted that I conform to those expectations simply out of respect for his/her authority.	○	○	○	○	○

30. Q30

	1	2	3	4	5
As I was growing up, if my parent made a decision in the family that hurt me, he/she was willing to discuss that decision with me and to admit it if he/she had made a mistake.	○	○	○	○	○

31. Do you think the way you were raised has influenced the parenting and discipline advice you give in clinic?

- ○ Yes
- ○ No
- ○ Unsure

Optional Comment:

Human Research Participant Protection
The University of Oklahoma
IRB APPROVED
AAHRPP
IRB NUMBER: 1477
IRB APPROVAL DATE: 11/24/2014
